# Environmental Supports, Expectations, and Barriers to Leisure‐Time Physical Activity During Pregnancy: A Rural–Urban Comparison

**DOI:** 10.1111/jrh.70166

**Published:** 2026-05-14

**Authors:** Carly Williamson Rogers, Alex Crisp, Katrina Wilhite, Alexis Thrower, Elly Marshall, J. P Marrero‐Rivera, Jacob B. Gallagher, Bethany Barone Gibbs, Kara M. Whitaker, Sharon E. Taverno Ross

**Affiliations:** ^1^ Department of Health and Human Development University of Pittsburgh Pittsburgh Pennsylvania USA; ^2^ Department of Health, Sport, and Human Physiology University of Iowa Iowa City Iowa USA; ^3^ Department of Epidemiology and Biostatistics West Virginia University Morgantown West Virginia USA; ^4^ Department of Exercise Science Seton Hill University Greensburg Pennsylvania the United States; ^5^ Department of Kinesiology Iowa State University Ames Iowa USA

**Keywords:** barriers, environmental supports, physical activity, pregnancy, rurality

## Abstract

**Background:**

Physical activity (PA) during pregnancy has well‐documented benefits, yet pregnant rural residents may face additional barriers to PA.

**Objective:**

To examine whether leisure‐time physical activity (LTPA) and correlates of LTPA, including exercise outcome expectations, barriers, and environmental supports for PA, differ by rurality, and explore whether rurality moderates the associations between these correlates and LTPA during pregnancy.

**Methods:**

Pregnant individuals from Iowa and West Virginia (*n* = 374) completed questionnaires assessing LTPA, exercise outcome expectations and barriers during each trimester, and environmental supports for PA at the first trimester. Rurality status was categorized as urban, micropolitan, or small town rural using Rural–Urban Commuting Area codes. Robust linear mixed effects models included trimester as a fixed effect and a random intercept for participant. Environmental supports were analyzed using a robust linear model restricted to first‐trimester data. Models were adjusted for site, age, pre‐pregnancy body mass index, education, parity, and minority status.

**Results:**

In unadjusted models, small‐town rural participants engaged in less LTPA than urban participants (9.02 vs. 12.14 MET‐h/week, *p* = 0.021), though this was not significant after adjustment. Small‐town rural participants reported fewer environmental supports for PA than urban participants (*β* = −0.42 SD, *p* = 0.002). Rurality did not moderate the associations between examined correlates and LTPA.

**Conclusions:**

Rurality was not associated with LTPA, exercise expectations, or barriers after adjustment. Although small‐town rural participants reported fewer environmental supports for PA, this was not associated with LTPA, warranting further investigation in larger samples to determine whether improving environmental supports would translate into increased LTPA during pregnancy.

## 1 Introduction

Pregnant individuals are recommended to engage in 150 min of moderate‐intensity aerobic physical activity per week during pregnancy and the postpartum period [1]. The benefits of engaging in this amount of physical activity include reduced risk of excessive gestational weight gain, gestational diabetes, preeclampsia, large for gestational age (LGA) infants, and postpartum depression, as well as shorter labor and faster recovery after birth [1]. However, in the United States, less than 25% of individuals meet physical activity recommendations during pregnancy [2]. Leisure‐time physical activity (LTPA), defined as physical activity done during an individual's free time [3], is a feasible target for physical activity interventions because it is modifiable and more closely tied to better health outcomes in adults than other domains of physical activity [3, 4, 5]; however, unique barriers arise during pregnancy that may prevent physical activity engagement. These barriers to LTPA participation during pregnancy include factors such as fatigue, discomfort, and nausea [6]. Some individuals may also have concerns based on outdated information that moderate or vigorous intensity physical activity during pregnancy may contribute to miscarriage, poor fetal growth, musculoskeletal injury, or premature delivery [7].

Individuals residing in rural areas engage in less physical activity compared to their urban counterparts [8]. The US Department of Agriculture uses measures of population density, urbanization, and commuting patterns to determine rurality, with rurality being characterized by lower population density, lesser urbanization, and reduced commuting to urban areas [9]. Rural residents face distinct barriers to engaging in physical activity, including limited access to exercise facilities, fewer places to be active, limited resources within the built and natural environment, and greater safety concerns, such as free ranging cats and dogs [10, 11]. However, it is uncertain how these challenges interact with the additional physical activity barriers experienced during pregnancy. It is possible that pregnant individuals in rural as compared to urban areas face greater challenges in meeting physical activity recommendations due to differences in exercise outcome expectations, perceived barriers, and available environmental supports, though these hypotheses are largely unstudied. Understanding these disparities is essential for developing targeted interventions that support physical activity during pregnancy across different geographic settings.

As such, the current study aimed to (1) determine whether LTPA differs among pregnant individuals residing in rural and urban communities and (2) examine whether environmental supports, exercise outcome expectations, and perceived barriers to PA differ by rurality. As an exploratory aim, this study also investigated whether rurality moderates the associations between these correlates and LTPA during pregnancy. We hypothesized that rural pregnant individuals would report lower LTPA, fewer environmental supports, and lower exercise outcome expectations, as well as greater perceived barriers to PA compared to their urban counterparts. We further hypothesized that environmental supports and exercise expectations would be positively associated with LTPA, while perceived barriers would be negatively associated with LTPA across all rurality groups, with stronger associations expected in urban environments.

## 2 Methods

### 2.1 Study Design and Setting

The multi‐site Pregnancy 24/7 cohort study (NCT04749849) was conducted at the University of Iowa, West Virginia University , and University of Pittsburgh. The purpose of the study was to investigate 24‐h movement behaviors (physical activity, sedentary behavior, and sleep) across pregnancy and their associations with hypertensive disorders of pregnancy and other adverse pregnancy outcomes. Additional details about the study are reported elsewhere [12]. The current study conducted a secondary analysis using data from two sites, University of Iowa  and West Virginia University. Data from the University of Pittsburgh site were excluded, as a minimal number of participants from that site were classified as residing in a rural area (*n* = 3) and rural–urban comparisons were our focus.

### 2.2 Participants

Eligibility criteria for the Pregnancy 24/7 study included an age range of 18 to 45 years and a gestational age of less than 13 weeks at enrollment [12]. Because the study aimed to identify optimal movement and sleep patterns related to lower risk of adverse pregnancy outcomes (e.g., hypertensive disorders of pregnancy and gestational diabetes), exclusion criteria encompassed the use of blood pressure‐ or glucose‐lowering medications, treatment for sleep disorders, severe mobility limitations, or other conditions that could obscure the study exposures and outcomes.

Recruitment methods for the University of Iowa included mass emails sent to university employees and patients, newsletter advertisements, and paper fliers in health care facilities and community areas. West Virginia University recruitment methods included direct messaging through a patient‐facing app to first‐trimester patients, paper and electronic flyers in West Virginia University Medicine buildings, and university newsletters. Participants provided informed consent during the first‐trimester assessment. All protocols and study materials were approved through a single IRB with the University of Iowa's Institutional Review Board (202002630).

### 2.3 Measures

#### 2.3.1 Leisure‐Time Physical Activity (LTPA)

Participants completed the Pregnancy Physical Activity Questionnaire (PPAQ) to measure their LTPA during each trimester of pregnancy. The PPAQ was developed to better understand the physical activities of pregnant individuals, including questions that ask the participant to report the amount of time spent engaging in 32 different activities within five overarching categories, including household/caregiving, occupational, sports and exercise, transportation, and inactivity [13]. The PPAQ is validated with demonstrated reliability in ethnically diverse populations of pregnant individuals, with a Cronbach's alpha of 0.89 [13]. Self‐reported responses to the sports and exercise category were used to estimate LTPA as total metabolic equivalents (MET) hours/week.

#### 2.3.2 Exercise Outcome Expectations and Barriers

Participants self‐reported their exercise outcome expectations and perceived barriers through an adapted Exercise Outcome Expectations and Barriers questionnaire during each trimester [14]. The questionnaire included 21 total items using a 5‐point Likert scale ranging from strongly disagree (1) to strongly agree (5). Twelve items, with a score ranging from 12 to 60, focused on expected outcomes when participating in LTPA, and nine items, with a score ranging from 9 to 45, focused on perceived barriers to participating in LTPA using an adapted version of the original 14‐item barriers subscale. The Exercise Outcome Expectations and Barriers questionnaire was validated and demonstrated reliability in a sample of middle‐class, full‐time employees, but has not yet been validated in a sample of pregnant individuals [14].

#### 2.3.3 Environmental Supports for Physical Activity

Participants provided information about environmental supports for physical activity through responses to the Environmental Supports for Physical Activity Questionnaire [15] at baseline; this was repeated if the participant changed their home address during the study. This instrument included 13 items assessing neighborhood access and barriers, with a score ranging from 0 to 48, and 13 items evaluating community access and barriers, with a score ranging from 0 to 40. These two scores were combined to create a total environmental supports score, with a maximum possible value of 88. Higher scores indicated greater environmental supports for physical activity. The Environmental Supports for Physical Activity Questionnaire was previously validated and demonstrated reliability in a sample of adults, but has not yet been validated in a sample of pregnant individuals [16].

#### 2.3.4 Rurality

Participants were categorized into one of three groups using Rural–Urban Commuting Area (RUCA) codes [9]. RUCA codes place counties within the United States on an urban–rural continuum based on the county's degree of urbanization and proximity to metropolitan areas [9]. Using the 2010 RUCA code database and participant addresses, groups were classified as metropolitan urban (RUCA 1‐3), micropolitan rural (RUCA 4‐6), or small‐town rural (RUCA 7‐10).

#### 2.3.5 Demographic Characteristics

Participants provided information about their demographic characteristics via a questionnaire at baseline, including race, ethnicity, education, age, and parity. Participants selected all that applied from the following categories: black/African American, Asian, Native Hawaiian/ Pacific Islander, or Native American. Participants self‐reported their ethnicity as Hispanic/Latino (yes/no). Participants who selected more than one racial category were classified as Multiple Race. Race and ethnicity were used to determine “Underrepresented Minority Status (URM),” which was defined as any person that identified as non‐white or Hispanic. Participants selected from the following categories for education: no high school degree, high school graduate/GED, some college, college graduate, or graduate degree. For parity, participants selected from the following options: no children, one child, or two or more children. Height was measured in duplicate or triplicate (if measures differed by >0.5 cm) during the first trimester study visit. Pre‐pregnancy weight was abstracted from medical records. Height and pre‐pregnancy weight were used to calculate pre‐pregnancy body mass index (BMI; kg/m^2^). This method has been found valid and reliable in pregnant populations [17, 18].

### 2.4 Statistical Analyses

Descriptive statistics were calculated for demographic information. Group differences across participant characteristics by rurality group were examined using robust linear models, chi‐square, and Fisher's exact test, as appropriate. Mean and standard deviations (SD) were calculated for LTPA, exercise outcome expectations, exercise barriers, and environmental support scores by trimester and rurality status.

Robust linear mixed‐effects models (robustlmm package, version 3.4‐2) were used to examine associations between rurality and LTPA, exercise outcome expectations, and exercise barriers. These models included trimester as a fixed effect and a random intercept for participant to account for repeated measures, therefore evaluating average differences across pregnancy rather than trimester‐specific differences. Environmental supports were analyzed using a robust linear model (MASS package, version 7.3‐65) restricted to first‐trimester data, as no participants changed rurality status across pregnancy. Scores from environmental supports, exercise outcome expectations, and exercise barriers were standardized prior to analysis using *z*‐score transformation to facilitate comparisons across instruments. Adjusted models included study site, age, pre‐pregnancy BMI, education, parity, and minority status as confounders. Robust estimation was applied to reduce the influence of outliers, group imbalance, and violations of normality identified during preliminary model diagnostics using the performance package (version 0.16.0).

As an exploratory aim, interaction terms between each standardized correlate and rurality were added to the adjusted models to examine whether rurality moderated the associations between each correlate and LTPA. Estimated marginal means and 95% confidence intervals were extracted from all models using the emmeans package (version 2.0.1), and rurality‐specific slopes and pairwise contrasts relative to the urban reference were estimated for the interaction models. Global tests of each model term were conducted using *F*‐tests. Statistical significance was set at *p* < 0.05, and all analyses were conducted in R (version 4.5.2).

## 3 Results

The Pregnancy 24/7 cohort enrolled 500 participants across three study sites. Data from the Pittsburgh site were excluded as nearly all participants resided in urban areas, leaving 375 participants from Iowa and West Virginia. One participant was excluded due to missing sociodemographic data, resulting in a final analytic sample of 374 participants.

Participant demographic characteristics for the full sample and by rurality group are presented in Table 1. Most participants lived in metropolitan urban areas (*n* = 264), with fewer in micropolitan rural (*n* = 42) and small‐town rural (*n* = 68). Micropolitan rural participants were younger on average compared to urban and small‐town rural participants, while small‐town rural participants had the highest pre‐pregnancy BMI and the lowest proportion with a graduate degree. Parity differed across groups, with urban participants most likely to have no previous children and small‐town rural participants more likely to have two or more previous births. Underrepresented minority status was highest in urban areas, compared to micropolitan rural and small‐town rural.

**TABLE 1:** Participant demographic characteristics, overall and by rurality.

	Total (*N* = 374)	Urban (*n* = 264)	Micro (*n* = 42)	Small town (*n* = 68)	*p*‐value
**Age, years**	30.2 (4.5)	30.4 (4.2)	28.0 (5.1)	30.6 (4.7)	**0.010**
**Pre‐pregnancy body mass index**	27.6 (6.5)	27.0 (5.7)	28.7 (8.4)	29.5 (7.6)	**0.023**
**Education level**					**<0.001**
Some college	90 (24.1%)	43 (16.3%)	19 (45.2%)	28 (41.2%)	
College graduate	128 (34.2%)	87 (33.0%)	11 (26.2%)	30 (44.1%)	
Graduate degree	156 (41.7%)	134 (50.8%)	12 (28.6%)	10 (14.7%)	
**Parity**					**<0.001**
None	160 (42.8%)	126 (47.7%)	16 (38.1%)	18 (26.5%)	
1	133 (35.6%)	95 (36.0%)	14 (33.3%)	24 (35.3%)	
2 or more	81 (21.7%)	43 (16.3%)	12 (28.6%)	26 (38.2%)	
**Underrepresented minority status (%)**	56 (15.0%)	48 (18.2%)	2 (4.8%)	6 (8.8%)	**0.022**
**Race**					0.095
Black/African American	9 (2.4%)	8 (3.0%)	0 (0%)	1 (1.5%)	
Asian	20 (5.3%)	19 (7.2%)	1 (2.4%)	0 (0.0%)	
White	334 (89.3%)	227 (86.0%)	41 (97.6%)	66 (97.1%)	
Multiple/Other	11 (2.9%)	10 (3.8%)	0 (0.0%)	1 (1.5%)	
**Ethnicity**					0.500
Hispanic/Latino	18 (4.8%)	12 (4.5%)	1 (2.4%)	5 (7.4%)	
**Study site**					**<0.001**
Iowa	250 (66.8%)	188 (71.2%)	13 (31.0%)	49 (72.1%)	
West Virginia	124 (33.2%)	76 (28.8%)	29 (69.0%)	19 (27.9%)	

*Note*: Mean (SD) or *n* (%). Rurality categorized using RUCA codes: urban (1–3), micropolitan (4–6), small‐town rural (7–10). Underrepresented minority status defined as non‐White. Participants selecting more than one race or another race not listed were categorized as Multiple/Other. Group differences tested using robust linear models, chi‐square (parity, minority status, study site), and Fisher's exact test (education, race, Hispanic ethnicity). Bold text indicates *p* < 0.05.

Table 2 presents mean values for LTPA, exercise outcome expectations, exercise barriers, and environmental supports scores by trimester and rurality group. Overall, LTPA mean values were highest in the first trimester and declined by the third trimester, with urban participants consistently reporting higher values than micropolitan and small‐town rural participants. All groups met the recommended guideline of 150 min of moderate to vigorous physical activity (∼10 MET hours/week) [1]. Exercise outcome expectations and barriers scores were similar across rurality groups and remained relatively stable throughout pregnancy. However, urban participants reported higher mean environmental supports scores compared to micropolitan and small‐town rural participants.

**TABLE 2:** Leisure‐time physical activity, exercise outcomes expectations, exercise barriers, and environmental supports by trimester and rurality group.

	Trimester 1				Trimester 2				Trimester 3			
	Overall *N* = 374	Urban *N* = 264	Micro *N* = 42	Rural *N* = 68	Overall *N* = 341	Urban *N* = 245	Micro *N* = 35	Rural *N* = 61	Overall *N* = 3335	Urban *N* = 244	Micro *N* = 31	Rural *N* = 60
**LTPA** **(MET h/week)**	14.0 (12.2)	14.9 (12.0)	11.5 (12.5)	12.2 (12.8)	14.1 (12.9)	15.4 (13.6)	10.2 (8.5)	11.0 (11.7)	10.8 (11.0)	11.0 (9.9)	11.3 (14.4)	10.0 (13.1)
**Exercise outcome expectations score**	46.8 (8.6)	47.3 (8.2)	44.2 (10.6)	46.6 (8.5)	46.0 (8.0)	46.5 (7.7)	43.6 (10.3)	45.5 (7.9)	45.8 (7.6)	46.2 (7.5)	43.6 (7.2)	45.5 (8.1)
**Exercise barriers score**	28.0 (5.7)	27.9 (5.4)	28.1 (6.0)	28.3 (6.5)	27.5 (5.7)	27.1 (5.4)	28.6 (6.4)	28.4 (6.3)	27.5 (5.7)	27.4 (5.6)	27.9 (6.0)	27.7 (6.1)
**Environmental supports score** ^a^	61.9 (12.5)	64.2 (12.0)	55.8 (11.3)	57.0 (12.5)		—	—	—	—	—	—	—

*Note*: Mean (SD). Exercise outcome expectations score: sum of 12 items (range 12–60); exercise barriers score: sum of 9 items (range 9–45); environmental supports score: range 0–88.

Abbreviations: LTPA, leisure‐time physical activity; MET, metabolic equivalent.

^a^Environmental supports were measured at the first trimester only.

Associations between rurality and each outcome are presented in Table 3. In unadjusted models, rurality was significantly associated with LTPA (*F* = 4.80, *p* = 0.008), with micropolitan (*β* = −3.28, *p* = 0.057) and small‐town rural participants (*β* = −3.12, *p* = 0.021) reporting lower LTPA than urban participants. After confounder adjustment, the association between rurality and LTPA was no longer significant (*F* = 1.02, *p* = 0.361). Pre‐pregnancy BMI (*F* = 12.94, *p* < 0.001), education (*F* = 6.12, *p* = 0.002), and URM (*F* = 5.90, *p* = 0.015) were independently associated with LTPA in the adjusted model.

**TABLE 3:** Associations between rurality and leisure‐time physical activity, exercise outcome expectations, exercise barriers, and environmental supports.

	Unadjusted			Adjusted		
	EMM [95% CI]	*β* (SE)	*p*‐value	EMM [95% CI]	*β* (SE)	*p*‐value
**LTPA** **(MET h/week)**						
Urban	12.14 [11.06, 13.23]	Ref		10.13 [8.67, 11.60]	Ref	
Micro	8.86 [6.10, 11.62]	−3.28 (1.51)	0.057	8.13 [5.29, 10.97]	−2.00 (1.50)	0.312
Small town	9.02 [6.87, 11.17]	−3.12 (1.23)	**0.021**	9.20 [6.82, 11.58]	−0.93 (1.23)	0.660
**Exercise outcome expectations** **(*z*‐score)**						
Urban	0.10 [−0.01, 0.20]	Ref		−0.10 [−0.24, 0.04]	Ref	
Micro	−0.21 [−0.47, 0.06]	−0.30 (0.15)	0.071	−0.10 [−0.37, 0.17]	−0.00 (0.14)	0.999
Small town	−0.01 [−0.22, 0.19]	−0.11 (0.12)	0.550	0.04 [−0.19, 0.26]	0.13 (0.12)	0.401
**Exercise barriers** **(*z*‐score)**						
Urban	−0.02 [−0.12, 0.08]	Ref		0.06 [−0.08, 0.20]	Ref	
Micro	0.10 [−0.16, 0.35]	0.12 (0.14)	0.621	0.04 [−0.23, 0.32]	−0.02 (0.15)	0.984
Small town	0.10 [−0.10, 0.30]	0.12 (0.11)	0.492	0.10 [−0.13, 0.33]	0.04 (0.12)	0.910
**Environmental supports** **(*z*‐score)** ^a^						
Urban	0.29 [0.18, 0.40]	Ref		−0.01 [−0.16, 0.14]	Ref	
Micro	−0.37 [−0.65, −0.09]	−0.66 (0.16)	**<0.001**	−0.22 [−0.51, 0.07]	−0.21 (0.15)	0.288
Small town	−0.31 [−0.53, −0.09]	−0.60 (0.13)	**<0.001**	−0.43 [−0.68, −0.19]	−0.42 (0.13)	**0.002**

*Note*: LTPA values in MET h/week; environmental supports, exercise outcome expectations, and exercise barriers are standardized (*z*‐score). Bolded values are statistically significant, *p*‐value < 0.05.

Abbreviation: EMM, estimated marginal mean.

^a^Environmental supports were measured at the first trimester only. Adjusted models include age, pre‐pregnancy BMI, education, parity, minority status, and study site as confounders.

Rurality was significantly associated with environmental supports in both unadjusted (*F* = 17.12, *p* < 0.001) and adjusted models (*F* = 5.87, *p* = 0.003). After adjustment, small‐town rural participants reported significantly fewer environmental supports than urban participants (*β* = −0.42 SD, 95% CI −0.68 to −0.19, *p* = 0.002), while micropolitan participants did not differ significantly (*β* = −0.21 SD, *p* = 0.288). Study site (*F* = 54.15, *p* < 0.001) and education (*F* = 7.88, *p* < 0.001) were also independently associated with environmental supports. Rurality was not significantly associated with exercise outcome expectations or exercise barriers in unadjusted or adjusted models (expectations: unadjusted *F* = 2.33, *p* = 0.097; adjusted *F* = 0.72, *p* = 0.489; barriers: unadjusted *F* = 0.74, *p* = 0.478; adjusted *F* = 0.07, *p* = 0.931).

**FIGURE 1:**
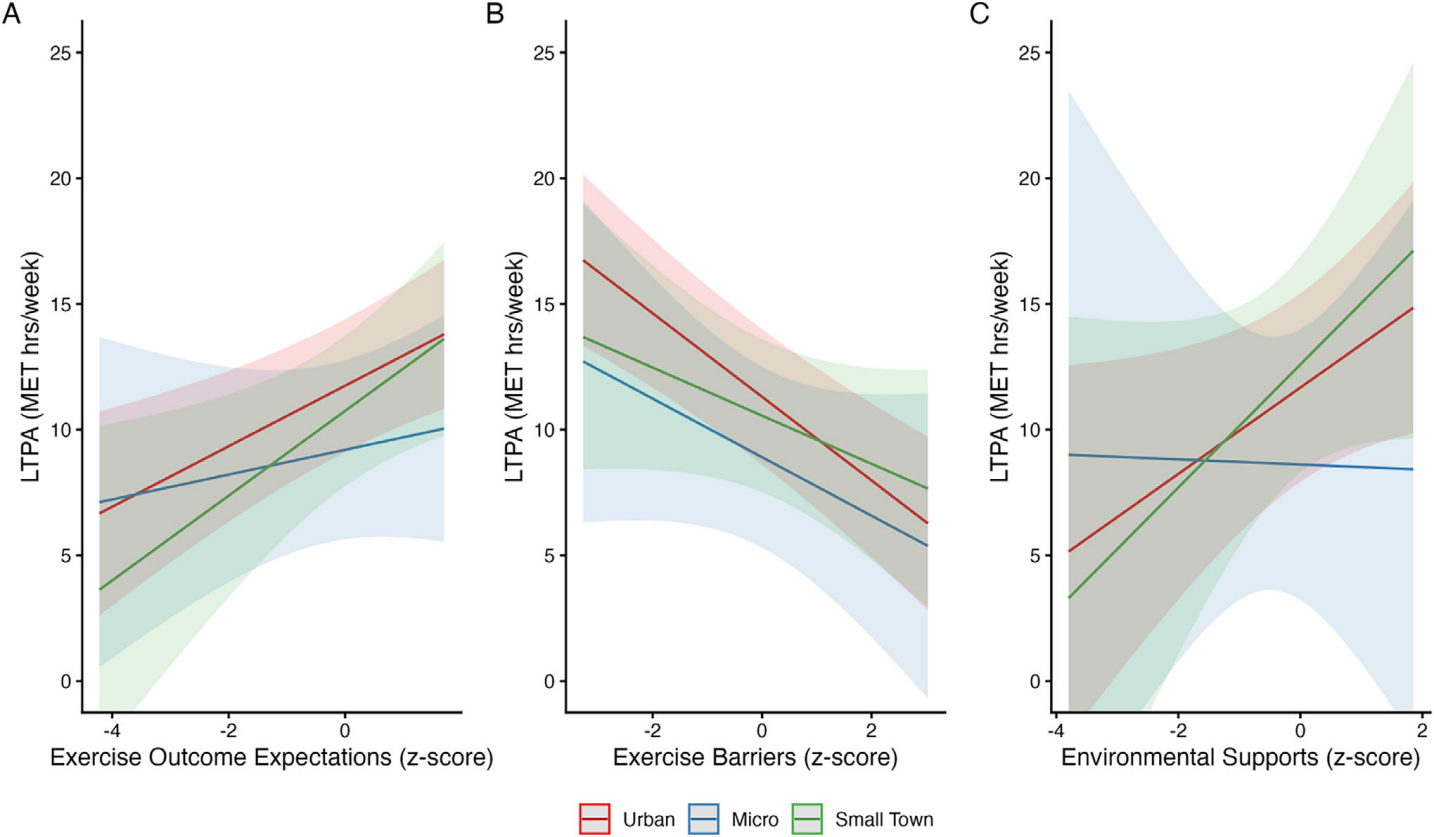
Associations between exercise outcome expectations (A), exercise barriers (B), and environmental supports (C) with leisure‐time physical activity (LTPA) during pregnancy, by rurality group. Predicted values are derived from adjusted robust linear mixed‐effects models (panels A and B) and a robust linear model (panel C), with all covariates held at their mean or reference values. Shaded areas represent 95% confidence intervals.

Results from the exploratory moderation analysis are presented in Figure 1 and Table S1. Exercise outcome expectations were positively associated with LTPA overall (*F* = 9.74, *p* = 0.002), with a slope of 0.15 MET‐h/week per SD increase among urban participants. Exercise barriers were negatively associated with LTPA overall (*F* = 11.81, *p* < 0.001), with a slope of −0.29 MET‐h/week per SD increase among urban participants. Environmental supports were not significantly associated with LTPA overall (*F* = 2.23, *p* = 0.136).

Rurality did not significantly moderate the association between any of the correlates and LTPA. The interaction between exercise outcome expectations and rurality was not significant (*F* = 0.71, *p* = 0.491), nor were the interactions for exercise barriers (*F* = 0.55, *p* = 0.580) or environmental supports (*F* = 0.50, *p* = 0.607). Slopes did not differ significantly between micropolitan or small‐town rural participants and urban participants for any instrument, suggesting that the associations between these correlates and LTPA are similar after adjustment for sociodemographic and individual‐level factors.

## 4 Discussion

Several important findings were revealed in our study. First, small‐town rural participants engaged in less LTPA than urban participants in unadjusted models, but this difference was no longer significant after confounder adjustment, suggesting that sociodemographic and individual‐level factors may account for observed rural–urban differences in LTPA. Second, small‐town rural participants reported significantly fewer environmental supports for PA compared to urban participants even after adjustment, while micropolitan participants did not differ significantly. Third, exercise outcome expectations and exercise barriers were independently associated with LTPA across all rurality groups, with higher expectations associated with greater LTPA and greater barriers associated with lower LTPA. Finally, rurality did not significantly moderate the associations between any of the correlates and LTPA, suggesting that the relationships between environmental supports, exercise expectations, barriers, and LTPA are similar regardless of geographic context. Below, we expand on these findings in more detail.

First, partially supporting our hypothesis, small‐town rural participants reported significantly lower LTPA compared to urban participants in the unadjusted models. However, although the direction of associations remained consistent, these differences were attenuated after adjustment for confounders. This highlights potential disparities in physical activity participation that may be attributed to underlying social or environmental factors, such as educational level. Prior work has shown that rural pregnant individuals often face additional barriers to their physical activity. For example, Newson et al. [19] found that rural pregnant individuals reported limited access to safe exercise facilities, fewer support systems, and being confused by targeted health messaging.

Second, and consistent with our hypothesis, small‐town rural pregnant individuals reported significantly lower environmental supports for physical activity compared to their urban counterparts. This association has been documented in the general population [20, 21], but its relevance to pregnant individuals has not been previously explored. These results suggest the need for environmental interventions for rural pregnant populations. Contrary to our hypothesis, exercise outcome expectations and barriers did not significantly differ by rurality group. This suggests that motivational and perceptual aspects of physical activity may be consistent across geographic settings, whereas environmental context may play a more influential role regardless of setting [20].

Third, our findings confirmed that greater exercise outcome expectations and lower exercise barriers were each associated with higher LTPA in adjusted models, which is consistent with previous findings [22, 23]. Conversely, environmental supports were not significantly associated with LTPA in any rurality group. These findings suggest that psychosocial factors may play a more prominent role in shaping LTPA during pregnancy than environmental influences. One possible explanation of this is that an individual's beliefs about the benefits of exercise and their perceptions of barriers may more directly influence behavioral decision‐making during pregnancy than environmental influences [24].

Lastly, the moderation analysis illustrated no rural–urban differences in the associations between potential correlates and LTPA, indicating that the mechanisms through which psychosocial and environmental factors relate to LTPA during pregnancy may operate similarly across geographical context. This contrasts with previous findings that suggested the mechanisms supporting LTPA differ by rurality [24, 25]. Though those studies were done in an adult population and were not pregnancy specific, suggesting that pregnancy may alter an individuals’ perception of psychosocial and environmental factors [24].

## 5 Strengths and Limitations

This study has several strengths. It is one of the first to examine differences in LTPA among pregnant individuals across levels of rurality, providing new insights into how geographic context may influence physical activity during pregnancy. The inclusion of multiple factors, such as exercise outcome expectations, barriers, and environmental supports, allows for a broader understanding of the determinants of LTPA. The use of longitudinal data allows for comparisons across pregnancy rather than a focus on trimester‐specific differences. Additionally, the use of validated instruments and a sample drawn from both urban and rural populations enhances the generalizability of findings.

However, this study is not without limitations. LTPA, exercise outcome expectations and barriers, and environmental supports were self‐reported, which may introduce recall or social desirability bias [26]. The Exercise Outcome Expectations and Barriers Questionnaire and Environmental Supports for Physical Activity Questionnaire are not yet validated in a sample of pregnant individuals, which may introduce measurement uncertainty in this population. While this study focused exclusively on LTPA, future studies should consider replicating this study while examining other domains of physical activity. The environmental supports measure, while validated, may not fully capture all objective aspects of the built environment that may impact physical activity behaviors, particularly in pregnant individuals. The classification of rurality, though based on established RUCA codes, may not reflect more nuanced community‐level differences that could influence physical activity, such as social norms [23]. Lastly, participant characteristics, such as higher educational attainment, low ethnic/racial minority representation, and geographical location may limit the generalizability of findings across different demographic subgroups.

## 6 Public Health Implications

These findings highlight key opportunities for improving physical activity in rural populations during pregnancy. Although rural–urban differences in LTPA were attenuated after accounting for individual‐level factors, the persistently lower environmental supports reported by small‐town rural participants illustrate a need for community‐level strategies that increase access to safe, convenient places to be active. Also, given that exercise expectations and perceived barriers were strongly related to LTPA across all rurality groups, behavioral approaches, such as tailored prenatal education and counseling, may be effective regardless of geographical context. Taken together, these results suggest that public health efforts should combine environmental improvements in rural areas with scalable psychosocial interventions to support physically active pregnancies.

## 7 Conclusions

This study provides important insights into the physical activity experiences of pregnant individuals. Rural participants (particularly small‐town rural) reported fewer environmental supports for physical activity compared to their urban counterparts. Greater exercise outcome expectations and lower barriers were associated with higher LTPA during pregnancy, suggesting an intervention targeting these areas would be strategic for *all* pregnant individuals. Contrary to our hypothesis, environmental supports were not significantly associated with LTPA, and rurality did not moderate the associations between potential correlates and LTPA. Taken together, our findings highlight the role of both environmental and individual‐level behavioral determinants (outcome expectations and perceived barriers) in shaping physical activity during pregnancy and suggest that improving overall supports across these domains may help promote physical activity during pregnancy.

## Funding

The Pregnancy 24/7 Cohort Study is supported by grant R01HL153095 from the National Heart, Lung, and Blood Institute (NHLBI).

## Conflicts of Interest

The authors declare no conflicts of interest.

## Supporting information




**Supporting File 1**: jrh70166‐sup‐0001‐tableS1.docx

## References

[1] U.S. Department of Health and Human Services , Physical Activity Guidelines for Americans, 2nd ed. (U.S. Department of Health and Human Services, 2018).

[2] K. R. Hesketh and K. R. Evenson , “Prevalence of US Pregnant Women Meeting 2015 ACOG Physical Activity Guidelines,” American Journal of Preventive Medicine 51, no. 3 (2016): e87–e89. 27544437 10.1016/j.amepre.2016.05.023 PMC4982752

[3] B. D'Souza , “Leisure‐time Physical Activity From the Standpoint of Public Health,” Journal of Yoga & Physical Therapy S7 (2021): 004, 10.35248/2157-7595.21.S7:004.

[4] D. Steinbach and C. Graf , “Leisure Time Physical Activity and Sedentariness,” in Encyclopedia of Public Health, ed. W. Kirch (Springer, 2008), 10.1007/978-1-4020-5614-7_1968.

[5] P. Coenen , M. A. Huysmans , and A. Holtermann , “Associations of Occupational and Leisure‐Time Physical Activity With All‐Cause Mortality: An Individual Participant Data Meta‐Analysis,” British Journal of Sports Medicine 58, no. 24 (2024): 1527–1538, 10.1136/bjsports-2024-108117. 39255999 PMC11671921

[6] Office of Disease Prevention and Health Promotion , Promoting Physical Activity During and After Pregnancy: Implementing the Move Your Way® Campaign With a Focus on Maternal Health, Health.gov News, Published 2021, Accessed April 23, 2025, https://health.gov/news/202105/promoting‐physical‐activity‐during‐and‐after‐pregnancy‐implementing‐move‐your‐way‐campaign‐focus‐maternal‐health.

[7] American College of Obstetricians and Gynecologists , Physical Activity and Exercise During Pregnancy and the Postpartum Period, Committee Opinion No. 804, ACOG, 2020, Accessed April 23, 2025, https://www.acog.org/clinical/clinical‐guidance/committee‐opinion/articles/2020/04/physical‐activity‐and‐exercise‐during‐pregnancy‐and‐the‐postpartum‐period.

[8] K. A. Matthews , J. B. Croft , and Y. Liu , “Health‐Related Behaviors by Urban‐Rural County Classification—United States, 2013,” Mmwr Surveillance Summaries 66, no. 5 (2017): 1–8. 10.15585/mmwr.ss6605a1 PMC5829834 28151923

[9] U.S. Department of Agriculture, Economic Research Service , Rural‐Urban Commuting Area Codes—Documentation, Updated May 8, 2025, Accessed April 23, 2025, https://www.ers.usda.gov/data‐products/rural‐urban‐commuting‐area‐codes/documentation.

[10] C. G. Abildso , S. M. Daily , and M. R. U. Meyer , “Environmental Factors Associated With Physical Activity in Rural US Counties,” International Journal of Environmental Research and Public Health 18, no. 14 (2021): 7688. 34300138 10.3390/ijerph18147688 PMC8307667

[11] A. Lepe , V. Kaplan , A. Arreaza , R. Szpanderfer , D. Bristol , and M. S. Sinclair , “Environmental Impact and Relative Invasiveness of Free‐Roaming Domestic Carnivores: A North American Survey of Governmental Agencies,” Animals 7, no. 10 (2017): 78, 10.3390/ani7100078. 29036923 PMC5664037

[12] K. M. Whitaker , M. A. Jones , and K. Smith , “Study Design and Protocol of the Multisite Pregnancy 24/7 Cohort Study,” American Journal of Epidemiology 193, no. 3 (2023): 415–425. 10.1093/aje/kwad208 PMC11484610 37939072

[13] L. Chasan‐Taber , M. D. Schmidt , D. E. Roberts , D. Hosmer , G. Markenson , and P. S. Freedson , “Development and Validation of a Pregnancy Physical Activity Questionnaire,” Medicine and Science in Sports and Exercise 36, no. 10 (2004): 1750–1756. 15595297 10.1249/01.mss.0000142303.49306.0d

[14] M. A. Steinhardt and R. K. Dishman , “The Reliability and Validity of Expected Outcomes and Barriers for Habitual Physical Activity,” Medicine and Science in Sports and Exercise 21, no. suppl (1989): S51. 10.1097/00043764-198906000-00011 2786559

[15] J. Reed and B. Ainsworth , “Perceptions of Environmental Supports on the Physical Activity Behaviors of University Men and Women: A Preliminary Investigation,” Journal of American College Health 56, no. 2 (2007): 199–204. 17967768 10.3200/JACH.56.2.199-208

[16] K. A. Kirtland , D. E. Porter , and C. L. Addy , “Environmental Measures of Physical Activity Supports: Perception Versus Reality,” American Journal of Preventive Medicine 24, no. 4 (2003): 323–331. 12726870 10.1016/s0749-3797(03)00021-7

[17] H. Inskip , S. Crozier , J. Baird , et al., “Measured Weight in Early Pregnancy Is a Valid Method for Estimating Pre‐Pregnancy Weight,” Journal of Developmental Origins of Health and Disease 12, no. 4 (2021): 561–569, 10.1017/S2040174420000926. 33046167

[18] D. Shin , H. Chung , L. Weatherspoon , and W. O. Song , “Validity of Prepregnancy Weight Status Estimated From Self‐Reported Height and Weight,” Maternal and Child Health Journal 18, no. 7 (2014): 1667–1674, 10.1007/s10995-013-1407-6. 24337814

[19] L. Newson , K. Bould , B. Aspin‐Wood , L. Sinclair , Z. Ikramullah , and J. Abayomi , “The Lived Experiences of Women Exploring Healthy Lifestyle, Gestational Weight Gain and Physical Activity Throughout Pregnancy,” Health Expectations 25 (2022): 1717–1729, 10.1111/hex.13514. 35514097 PMC9327828

[20] M. E. Wende , E. W. Stowe , and J. M. Eberth , “Spatial Clustering Patterns and Regional Variations for Food and Physical Activity Environments Across the United States,” International Journal of Environmental Health Research 31, no. 8 (2021): 976–990. 31964175 10.1080/09603123.2020.1713304

[21] J. X. Fan , M. Wen , and N. Wan , “Built Environment and Active Commuting: Rural‐Urban Differences in the United States,” SSM—Population Health 3 (2017): 435–441. 29124104 10.1016/j.ssmph.2017.05.007 PMC5673263

[22] R. McKeough , C. Blanchard , and H. Piccinini‐Vallis , “Pregnant and Postpartum Women's Perceptions of Barriers to and Enablers of Physical Activity During Pregnancy: A Qualitative Systematic Review,” Journal of Midwifery & Womens Health 67, no. 4 (2022): 448–462, 10.1111/jmwh.13375. 35621324

[23] A. L. Harrison , N. F. Taylor , N. Shields , and H. C. Frawley , “Attitudes, Barriers, and Enablers to Physical Activity in Pregnant Women: A Systematic Review,” Journal of Physiotherapy 64, no. 1 (2018): 24–32, 10.1016/j.jphys.2017.11.012. 29289592

[24] C. A. Pelletier , N. White , A. Duchesne , and L. Sluggett , “Barriers to Physical Activity for Adults in Rural and Urban Canada: A Cross‐Sectional Comparison,” SSM—Population Health 16 (2021): 100964, 10.1016/j.ssmph.2021.100964. 34841038 PMC8606540

[25] C. Müller , L. Paulsen , J. Bucksch , et al., “Built and Natural Environment Correlates of Physical Activity of Adults Living in Rural Areas: A Systematic Review,” International Journal of Behavioral Nutrition and Physical Activity 21 (2024): 52, 10.1186/s12966-024-01598-3. 38702772 PMC11067138

[26] A. Althubaiti , “Information Bias in Health Research: Definition, Pitfalls, and Adjustment Methods,” Journal of Multidisciplinary Healthcare 9 (2016): 211–217, 10.2147/JMDH.S104807. 27217764 PMC4862344

