# Supplementary material for: Environmental Supports, Expectations, and Barriers to Leisure‐Time Physical Activity During Pregnancy: A Rural–Urban Comparison
Source: J Rural Health. 2026 May 14;42:e70166. doi: 10.1111/jrh.70166 (PMC13176539; doi:10.1111/jrh.70166)
Supplement: Supplementary file 1 — Supporting File 1: jrh70166‐sup‐0001‐tableS1.docx [file JRH-42-0-s001.docx]

**Supplemental Table 1: Moderation of Rurality on Associations between Exercise Outcomes Expectation, Exercise Barriers, Environmental Supports, and Leisure Time Physical Activity**

|  | Slope  [95% CI] | Δ β vs. Urban  (SE) | *p*-value |
| --- | --- | --- | --- |
| **Exercise Outcome Expectations**  **(Z-Score)** |  |  |  |
| Urban | 0.15  [0.06, 0.24] | Ref |  |
| Micro | 0.06  [-0.11, 0.23] | -0.09  (0.10) | 0.582 |
| Small-Town | 0.21  [0.04, 0.38] | 0.06  (0.10) | 0.759 |
| **Exercise Barriers**  **(Z-Score)** |  |  |  |
| Urban | -0.29  [-0.41, -0.17] | Ref |  |
| Micro | -0.20  [-0.49, 0.08] | 0.09  (0.16) | 0.788 |
| Small-Town | -0.17  [-0.39, 0.05] | 0.12  (0.13) | 0.522 |
| **Environmental Supports**  **(Z-Score)** † |  |  |  |
| Urban | 0.13  [0.00, 0.27] | Ref |  |
| Micro | -0.01  [-0.33, 0.31] | -0.14  (0.17) | 0.624 |
| Small-Town | 0.19  [-0.04, 0.42] | 0.06  (0.13) | 0.860 |

NOTE: Slope represents the expected change in LTPA (MET hrs/week) per 1 SD increase in the instrument score within each rurality group. Δβ represents the difference in slope compared to Urban (reference). † Environmental Supports were measured at the first trimester only.
